# The MUC1 Cytoplasmic Tail and Tandem Repeat Domains Contribute to Mammary Oncogenesis in FVB Mice

**DOI:** 10.4137/bcbcr.s655

**Published:** 2008-04-17

**Authors:** Christine L. Hattrup, Judy M. Bradley, Kari L. Kotlarczyk, Cathy S. Madsen, Joseph G. Hentz, Ronald J. Marler, Sandra J. Gendler

**Affiliations:** 1 Department of Biochemistry and Molecular Biology; 2 Department of Molecular Pharmacology and Experimental Therapeutics, Mayo Clinic College of Medicine, Mayo Clinic Arizona, 13400 E. Shea Boulevard, Scottsdale, Arizona, U.S.A. 85259; 3 Current Address: Department of Laboratory Medicine, Mayo Clinic Rochester, 200 First Street SW, Rochester, Minnesota, 55905, U.S.A

**Keywords:** MUC1, breast cancer, mouse models, cytoplasmic tail, tandem repeat, mucin

## Abstract

**Background:**

Though the importance of the transmembrane mucin MUC1 in mammary oncogenesis has long been recognized, the relative contributions of the cytoplasmic tail and tandem repeat domains are poorly understood.

**Methods:**

To address this, mouse models of mammary carcinogenesis were created expressing full-length, cytoplasmic tail-deleted, or tandem repeat-deleted MUC1 constructs.

**Results:**

Overexpression of full-length MUC1 resulted in tumor formation in young mice (≤12 months); however, loss of either the cytoplasmic tail or the tandem repeat domain abrogated this oncogenic capacity. Aged mice in all strains developed late-onset mammary tumors similar to those previously described for the FVB background.

**Conclusions:**

This study is the first spontaneous cancer model to address the relative importance of the cytoplasmic tail and tandem repeat domains to MUC1-driven mammary oncogenesis, and suggests that both of these domains are essential for tumor formation.

## Introduction

MUC1 is a tumor antigen and oncogene that is overexpressed in the majority (>90%) of human breast carcinomas (Gendler, 2001). Mouse models have been integral to the current understanding of the role of MUC1 in the development and progression of cancer of the mammary epithelium (note that the human homolog is written MUC1 and the mouse Muc1). Crossing the Muc1 knockout mouse (Muc1^−/−^) with strains expressing mouse mammary tumor virus (MMTV)-driven oncogenes suggests that Muc1 cooperates with other tumorigenic factors in cancer: MMTV-Wnt-1 and MMTV-TGFα mice show significantly delayed onset of tumors when on the Muc1^−/−^ background (Schroeder et al. 2003; Pochampalli et al. 2007), while MMTV-PyV MT (polyomavirus middle T antigen) mice lacking Muc1 have significantly reduced tumor progression with a trend towards decreased metastasis (Spicer et al. 1995). In complement to this, MUC1 has been shown to transform rat 3Y1 fibroblasts (Li et al. 2003), and MMTV-MUC1 transgenic mice develop spontaneous mammary tumors, indicating that MUC1 is an oncogene in its own right (Schroeder et al. 2004).

It is not yet clear what regions of the MUC1 protein are most involved in tumorigenesis. MUC1 is expressed on the cell surface as a heterodimer, comprised of two very different subunits (Gendler, 2001). The larger fragment is wholly extracellular, and is comprised mainly of a variable number of tandem repeats (TR) rich in serines and threonines. This subunit is heavily glycosylated; the majority of the size and glycosylation of the extracellular fragment is provided by the TR. The glycosylation is heterogeneous and differs in each tissue and physiologic state of the tissue (Hollingsworth and Swanson, 2004; Hattrup and Gendler, 2007). The extracellular subunit and in particular the TR domain are thought to play important roles in modulating cellular adhesion: bacteria, viruses, selectins, flagellin, and intercellular adhesion molecule-1 (ICAM-1) bind to the extracellular domain, while the enormous size and extended structure of MUC1 provide steric hindrance to cellular contact (reviewed in (Baldus et al. 2004; Hattrup and Gendler, 2007)).

The smaller MUC1 subunit is made up of a short extracellular stem, a single-pass transmembrane domain, and the 72-amino acid cytoplasmic tail (CT domain). This fragment is most notable for the interaction of the CT with numerous signaling proteins, many of which have been implicated in cancer (reviewed in (Singh and Hollingsworth, 2006; Hattrup and Gendler, 2007)). Among the proteins known to bind to MUC1 are several of the src family of kinases (Li et al. 2001; Mukherjee et al. 2005), the erbB receptor tyrosine kinases (Schroeder et al. 2001; Li et al. 2001), β-catenin (Yamamoto et al. 1997; Schroeder et al. 2003) and glycogen synthase kinase 3β (Li et al. 1998). Interaction with these important signaling networks is thought to facilitate MUC1-mediated oncogenesis. In addition, the CT has been found in the nucleus in association with potent transcription factors, including β-catenin (Wen et al. 2003; Huang et al. 2003), p53 (Wei et al. 2005), FOXO3a (Yin et al. 2004), estrogen receptor α (Wei et al. 2006) and affects nuclear localization (Thompson et al. 2006) and activation (Ahmad et al. 2007) of NF-κB, indicating that MUC1 may alter transcription in addition to its effects on signaling networks.

Despite acknowledgement of their very different functional roles, little has been done in physiological settings such as animal models to discern the relative importance of the TR and CT domains in MUC1 oncogenic activity. A recent paper (Kohlgraf et al. 2003) highlights the importance of proper model systems for studying the role of MUC1: *in vitro*, deletion of either the TR or the CT resulted in decreased invasion of transfected pancreatic cancer cells as compared to cells expressing full-length MUC1. *In vivo*, however, the same cells behaved quite differently. Those transfected with full-length MUC1 showed the least propensity to invade blood vessels and metastasize to the lymph nodes, while loss of either the TR or the CT restored the aggressive phenotype of the parental cells. Clearly, though much work on MUC1 function has been performed in cell culture, such *in vitro* systems fail to fully recapitulate the complex regulation of MUC1 function that is present *in vivo*. To further study the role of specific MUC1 domains in spontaneous mammary oncogenesis, we examined the effect of deleting the CT or TR domains on MUC1-induced tumorigenesis.

## Materials and Methods

FVB mice from Jackson Laboratory were bred to express CT-deleted (MUC1ΔCT) or TR-deleted (MUC1ΔTR) MUC1 under the MMTV LTR promoter (Schroeder et al. 2001). These strains were then crossed onto the Muc1^−/−^ knockout background (Spicer et al. 1995) and thus express only transgenic, human MUC1; crosses are called MMFK (MMTV-MUC1 × Muc1^−/−^), ΔCTK (MMTV-MUC1ΔCT × Muc1^−/−^) and ΔTRK (MMTV-MUC1ΔTR × Muc1^−/−^). The MMF mice are MMTV-MUC1 transgenics with endogenous Muc1. Two lines each of the various transgenic mice were created; both lines of MMF mice were originally characterized as developing tumors (Schroeder et al. 2004). For the purposes of this study, a single line of each transgenic was followed. The Muc1^−/−^ mice are fully congenic with the FVB background, having undergone greater than 20 backcrosses to FVB mice; the various transgenic mice were generated and have been continuously maintained in the FVB background. The number of mice in each group was: FVB wildtype controls (n = 20), Muc1^−/−^controls (n = 16), MMF (n = 38), MMFK (n = 22), ΔCTK (n = 19), and ΔTRK (n = 17). As the comparison of MMF mice to FVB wildtype controls has already been published (Schroeder et al. 2004), we focus here primarily on the transgenic mice on the Muc1^−/−^ background and include MMF results as a positive control.

Mice were permitted to breed continuously until 6–7 months of age and were monitored for tumor growth. At sacrifice (when tumors had reached dimensions of approximately 5–8 mm^2^), pituitary glands, mammary glands #2/3 and #4, and all tumors present were fixed in formalin; tumors and tissues were stained with hematoxylin and eosin and sections (mammary glands, tumors if present and pituitary glands) from every animal were analyzed by a veterinary pathologist. Tumor-free survival was analyzed according to the Kaplan-Meier method.

## Results and Discussion

Transgenic mice expressing full-length, CT-deleted, and TR-deleted MUC1 under the control of the MMTV promoter have been previously described (Schroeder et al. 2001; Schroeder et al. 2004); however, earlier studies were performed using animals with wildtype mouse Muc1 expression, potentially confounding analysis of the importance of the domains of the human protein. For this reason, the MUC1 transgenic mice were crossed onto the mouse Muc1^−/−^ background to examine the influence of the human MUC1 domains in isolation. These crosses are called MMFK (expressing full-length human MUC1), ΔCTK (CT-deleted human MUC1), and ΔTRK (TR-deleted human MUC1), all of which lack mouse Muc1.

Mice were observed for mammary tumor formation, along with FVB (wildtype, i.e. Muc1^+/+^) and Muc1^−/−^ controls. A comparison of MMF mice to FVB wildtype controls (i.e. overexpression of human MUC1 in addition to normal mouse Muc1 levels) has already been published (Schroeder et al. 2004), thus we focus here on the knockout crosses and include MMF results primarily as a positive control. Female mice were bred continuously for 6–8 months and then aged for tumor formation. The number of mice in each group was as follows: FVB (n = 20), Muc1^−/−^ (n = 16), MMF (n = 38), MMFK (n = 22), ΔCTK (n = 19), and ΔTRK (n = 17).

All mice in this study were on the FVB background. Two recent studies (Wakefield et al. 2003; Nieto et al. 2003) raised concerns about use of the FVB mice as tumor models: by 13 months, 40% of virgin FVB females developed spontaneous mammary hyperplasia, while 67% of multiparous animals had both mammary tumors and pituitary adenomas by age 18–23 months. Although it is not yet clear whether this phenotype is present in all strains of FVB mice, we were also able to detect late-onset mammary and pituitary abnormalities in our mice, starting at 13 months. As our published study (Schroeder et al. 2004) included mice that had been aged for 18 months, we felt that it was important to verify that MUC1 did indeed induce mammary gland tumors before FVB-induced tumors confounded the results. For this reason, data were analyzed both for overall survival and at age 12 months to address the influence of late-onset mammary tumor formation in the FVB strain.

**Figure 1.**
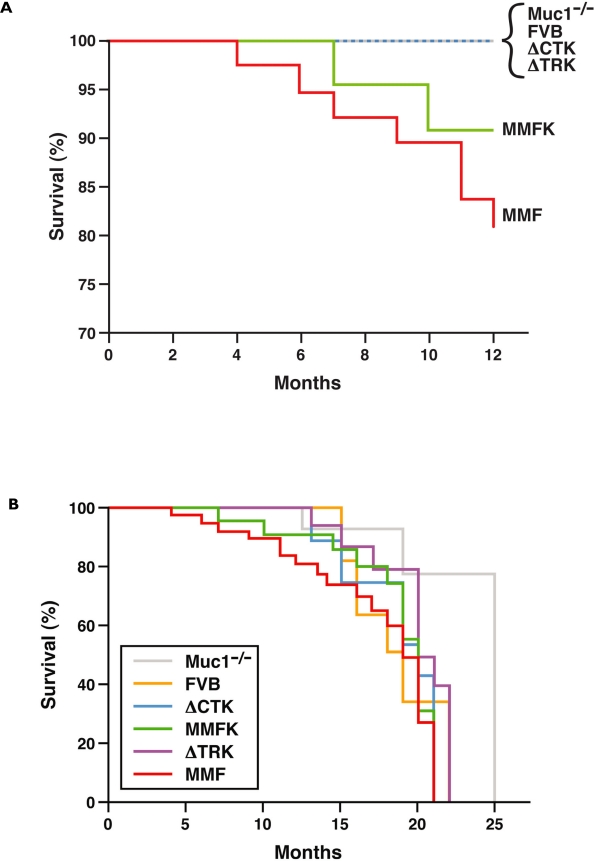
Tumor-free survival in MUC1 transgenic mice. Tumor-free survival was plotted according to the Kaplan-Meier method for (**a**) all lines (transgenic and control), up to 12 months of age and (**b**) until morbidity.

Figure 1A shows the Kaplan-Meier survival plot for all arms of the study through 12 months of age. Although no FVB or Muc1^−/−^ mice developed tumors by 12 months of age, both MMF and MMFK strains showed spontaneous mammary tumor formation within the first year (Fig. 1A). The difference in survival between MMFK and Muc1^−/−^ became statistically significant when mice were followed until tumor formation or morbidity (p = 0.03, Fig. 1B), similar to the results previously reported in MMF mice retaining mouse Muc1(Schroeder et al. 2004). Thus, these data confirm that human MUC1 overexpression in the mammary gland results in mammary tumorigenesis, regardless of the presence or absence of mouse Muc1.

Interestingly, MMFK mice develop tumors more slowly than do their MMF counterparts, which express the same full-length human transgene but retain mouse Muc1. Although the trend was not statistically significant, this finding agrees with previous studies showing reduced tumor formation or progression in mouse mammary tumor models in Muc1^−/−^ animals (Spicer et al. 1995; Schroeder et al. 2003; Pochampalli et al. 2007). This suggests that the transgenic human MUC1 behaves similarly to other oncogenes, which show reduced tumorigenic ability when mouse Muc1 is absent.

No mammary tumors were found by 12 months in either strain expressing domain-deleted MUC1 (ΔCTK and ΔTRK). This is despite similar expression levels of the MUC1 transgene in MMFK, ΔCTK, and ΔTRK animals (Schroeder et al. 2001). These results suggest that both the adhesion-modulating TR and cell-signaling CT domains are required for MUC1driven oncogenesis. The CT is clearly important in signal transduction; it interacts with a number of kinases (among them, src, EGFR, erbB-2,-3,-4, GSK3β, PKCδ, PI3-K), signaling (Grb2) and adhesion proteins (β-catenin) reviewed in (Singh and Hollingsworth, 2006; Hattrup and Gendler, 2007). Tumors from mice with Muc1 compared to those lacking Muc1 show increased β-catenin interactions, reduced apoptosis, decreased expression of activated ERK1/2, and increased association of kinases with their downstream signaling partners, suggesting that MUC1 likely serves as an adaptor protein to coordinate the signaling activites of these oncogenic proteins (Al Masri and Gendler, 2005). The functional role of the extracellular TR domain is more difficult to understand. MUC1 exists as a heterodimeric structure held together by non-covalent interactions. The functional importance of this structure is not clear, but presumably would enable rapid dissociation of the heavily glycosylated domain at the cell surface, which may relay information to the cell regarding conditions outside the cell. What these conditions are in oncogenesis remain unclear at the present time. The display of diverse carbohydrate structures on Muc1 likely serve to bind most bacteria, viruses and inhaled particles (in the case of the lung), thus mediating rapid clearance. The mucin domain may also bind growth factors, cytokines and other proteins that may function in as yet undefined ways similar to what has been observed for proteoglycans (Mahtouk et al. 2006). An additional consideration may be that the altered glycosylation of the TR or the sloughing of the extracellular domain is involved in inducing tolerance or evasion of immune surveillance, thus hindering early tumor growth when MUC1 TR expression is lost. Failure of the ΔTR mice to form tumors suggests an important role for this domain in oncogenesis. Future studies will be needed to define this key role, which will be confounded by the diverse nature of the carbohydrate structures which are known to vary in different tissues and physiological states.

Notably, when mice were permitted to age beyond 12 months, the incidence of mammary tumors in all arms (including Muc1^−/−^ and FVB negative control animals) increased rapidly (Fig. 1B). Beyond the hyperplasia reported previously (Wakefield et al. 2003), we observed frank tumors forming in the mammary gland beginning at 13 months (Fig. 1B). These tumors arose earlier than the age reported by Wakefield et al. for tumor formation in multiparous mice, where 4/6 (67%) mice aged 18–23 months were reported to develop mammary tumors. Muc1^−/−^ mice appear to develop tumors more slowly than FVB, although the difference is not significant (p = 0.16); this may reflect a role for mouse Muc1 in spontaneous FVB oncogenesis.

At sacrifice, mammary and pituitary glands were taken from multiparous female mice, formalin fixed, and embedded for histology. The pituitary adenomas described by Wakefield et al. (2003) were detected in all transgenic and control lines in this study. Of the subset of mice (n = 97) that reached age 13–25 months and were able to be assessed for pituitary adenomas, 32 (33.0%) had pituitary adenomas at time of sacrifice. This incidence is notably lower than the 67% (4 of 6) reported by Wakefield et al. However, considerably more aged mice were included in this study compared to that report (n = 97 vs. n = 6); in addition, mice were sacrificed earlier in this study (beginning at 13 months vs. beginning at 18 months) (Wakefield et al. 2003), perhaps precluding formation of frank pituitary adenomas in some mice. In the 97 mice examined for both pituitary and mammary abnormalities, the overall incidence of mammary tumors in aged, multiparous mice in this study is similar (52 of 97, 53.6%) to the 67% reported in Wakefield et al. (2003).

**Figure 2.**
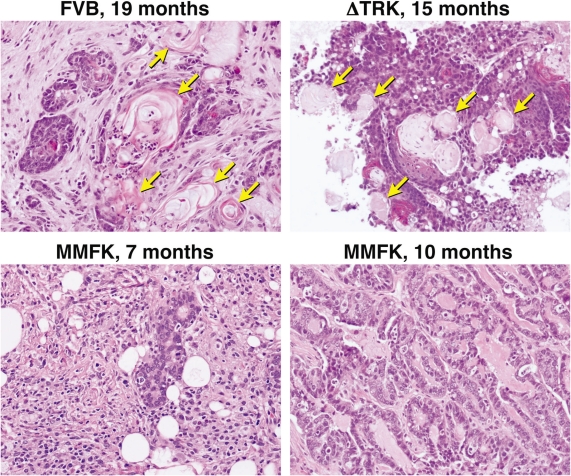
Histology of mouse tumors. Representative images (taken at 200X) are shown of mammary tumors present in: *upper left* a 19-month-old FVB, *upper right* a 15-month-old ΔTRK, *lower left* a 7-month-old MMFK, and *lower right* a 10-month-old MMFK. *Arrows* in the upper two panels denote the characteristic “keratin whorl” pattern observed in late-onset tumors.

We noted, however, that the tumors arising in young (≤12 mo), MUC1-transgenic mice (MMF or MMFK) did not resemble the late-onset FVB tumors histologically (Fig. 2). Late-onset FVB tumors (Fig. 2, *upper left*) displayed the squamous metaplasia and inflammatory infiltrates described by Wakefield; these were absent from the MUC1-driven tumors in young mice (Fig. 2, *lower panels*). Also visible in the FVB tumors was a characteristic “keratin whorl” pattern (*arrows*) that did not appear in the MUC1-driven tumors that arose prior to 12 months of age. Of note, the tumors arising in aged ΔCTK and ΔTRK mice more closely resembled the FVB tumors, with prominent keratin whorls, squamous metaplasia, and inflammatory infiltrates (Fig. 2, *upper right*). Most late-onset (>12 mo) tumors in MMF and MMFK mice also showed this morphological pattern (data not shown).

The characteristic histology and late onset of tumors arising in ΔCTK and ΔTRK mice suggests that these mammary tumors are related to the spontaneous, late-onset oncogenesis of the FVB background in aged mice. In contrast, the MMF and MMFK tumors arose earlier and had very different morphology, forming glandular and cribiform structures typical of adenocarcinoma. Together, these findings indicate that the ΔCTK and ΔTRK strains do not undergo MUC1-driven oncogenesis, suggesting that both of these domains are essential for its tumorigenic capacity in the mammary gland.

This study was the first spontaneous tumor model to examine the influence of the major functional domains of the MUC1 oncoprotein. *In vitro* studies and injected tumor models have provided contradictory evidence as to the relative importance and nature of the TR (adhesionmodulating) and CT (cellular signaling) functions. Given the wide range of potentially oncogenic factors that interact with the CT domain, it is not surprising to find that its deletion impairs MUC1-driven mammary tumorigenesis. However, it is somewhat more unexpected that the loss of the extracellular TR region resulted in no tumors; this suggests a need for further investigation into the mechanism by which the TR affects oncogenesis.

In summary, we report here that deletion of either the extracellular tandem repeats or the cytoplasmic tail of the MUC1 oncogene ablates MUC1-induced tumor formation in the mouse mammary gland prior to 12 months of age. Beyond 12 months, analysis of the role of MUC1 functional domains is confounded by the phenotype of spontaneous, late-onset mammary tumors arising in FVB mice, which was previously reported and confirmed herein using mice from a different source laboratory. This study is the first to analyze the relative importance of the MUC1 TR and CT domains in a spontaneous tumor model, and suggests that both the adhesive and signaling functions of MUC1 are important for its oncogenic potential.

## Abbreviations

CT: MUC1 cytoplasmic tail;
ΔCTK: CT-deleted MUC1 on Muc1^−/−^ strain;
MMF: MMTV-driven full-length MUC1 on Muc1^+/+^ strain;
MMFK: MMTV-driven full-length MUC1 on Muc1^−/−^ strain;
MMTV: mouse mammary tumor virus promoter;
TR: MUC1 tandem repeat;
ΔTRK: TR-deleted MUC1 on Muc1^−/−^strain.
